# Current approach to the diagnosis of sarcopenia in cardiovascular diseases

**DOI:** 10.3389/fnut.2024.1422663

**Published:** 2024-11-15

**Authors:** Xu Han, Gao Sen Zhang, Qi Rui Li, Zhen Zhang

**Affiliations:** Department of Ultrasound, The First Hospital of China Medical University, Shenyang, China

**Keywords:** cardiology, diagnosis, sarcopenia, skeletal muscle mass, cardiovascular diseases

## Abstract

Muscle wasting syndrome, also known as sarcopenia, is an age-related geriatric condition characterized by a gradual loss of muscle mass, strength, and function. Sarcopenia can be classified into primary and secondary types. Primary sarcopenia is primarily associated with aging, while secondary sarcopenia is caused by systemic diseases such as cancer, diabetes, liver cirrhosis, musculoskeletal disorders, and disuse changes. In recent years, increasing evidence suggests that cardiovascular diseases can promote the occurrence of sarcopenia through various pathophysiological mechanisms. Additionally, sarcopenia increases the risk of adverse outcomes in patients with cardiovascular disease such as rehospitalization and mortality. Therefore, screening and diagnosing sarcopenia are particularly important for patients with cardiovascular diseases. This article provides a brief overview of the research progress on diagnostic methods for sarcopenia in patients with cardiovascular diseases.

## 1 Introduction

The initial definition of sarcopenia was proposed by Irwin Rosenberg in 1989, referring to the age-related decrease in muscle mass and strength (1). In 2010, the European Working Group on Sarcopenia in Older People (EWGSOP) reached a consensus that defined it as an age-related syndrome characterized by decreased muscle mass, reduced muscle strength, and/or a decline in physical function (2). Later on, the Asian working group for sarcopenia (AWGS) published their own consensus on sarcopenia in Asia (3). Sarcopenia is associated with physical disabilities, a decreased quality of life, and an increased risk of mortality (4). In 2016, muscular dystrophy was officially recognized as a muscle disease and the ICD-10-MC diagnostic code was released (5). According to the EWGSOP standards, the prevalence of sarcopenia ranges from 8 to 36% in individuals under 60 years old and between 10 and 27% in those aged 60 and above (6). Cardiovascular diseases (CVD) include hypertension, coronary artery disease (CAD), acute myocardial infarction, arrhythmia, cardiomyopathy, valvular heart disease, congenital cardiovascular disease, and heart failure (HF). They are the leading cause of death and disability worldwide (7). Approximately 17.8 million people die from CVD each year, which accounts for one-third of global deaths (8). Myocardial infarction is present in many types of CVD (9). The prevalence of sarcopenia among patients with various CVD varies from 10.1 to 68.9% (10). Sarcopenia has emerged as a frequent complication in individuals suffering from CVD, and reduced muscle mass independently contributes to the risk of mortality associated with such conditions (11), thereby significantly impacting both the quality of life and prognosis for these patients (12, 13). Therefore, in the clinical diagnosis and treatment of CVD patients, accurately assessing and intervening in muscle wasting is becoming increasingly important.

## 2 The relationship between cardiovascular disease and sarcopenia

Sarcopenia is closely related to cardiovascular diseases. They interact with each other to accelerate the process of the disease. Sarcopenia and CVD share common pathogenesis, including hormonal changes, immunosenescence, impaired autophagy, oxidative stress, and mitochondrial dysfunction. The degree of muscle loss can be exacerbated by a sedentary lifestyle, prolonged bed rest, smoking and alcohol intake and obesity, and these factors are well established risk factors for cardiovascular disease.

Lack of physical activity, changes in body hormones, inadequate intake of nutrients, and imbalances in protein synthesis and utilization can easily lead to damage to mitochondrial structure and function as well as increased oxidative stress. Ultimately, this can result in the development of sarcopenia in patients with HF (14, 15). The prevalence of elderly patients with HF combined with sarcopenia is as high as 31%, and it is associated with reduced exercise capacity, poor quality of life, and adverse outcomes (16).

Hypertension and sarcopenia share similar underlying biological mechanisms, namely, low-grade chronic systemic inflammation. Hypertension is currently recognized as an inflammation-related disease. Many studies have found that proinflammatory cytokines such as CRP (C-reactive protein), IL-6 (interleukin 6), and TNF-*α* (tumor necrosis factor-alpha) increase abnormally in hypertensive patients (17, 18). In addition, chronic inflammation can accelerate protein breakdown and promotes sarcopenia by activation of the ubiquitin proteosome cascade (19). Accordingly, sarcopenia is common among adults with hypertension; the prevalence of sarcopenia among patients with hypertension ranges from 20.2 to 25.8%, which is significantly higher compared to the general population (20, 21).

Coronary artery disease (CAD) interacts with and influences sarcopenia. CAD promotes the occurrence of sarcopenia, as indicated by a meta-analysis that shows the prevalence of sarcopenia in CAD patients to be approximately 22.3% (10), and it is an independent risk factor for poor prognosis in CAD patients (22, 23). Sarcopenia can also contribute to the occurrence of CAD, as it results in a reduction in muscle mass and an increase in relative fat content caused by the substitution of muscle cells with adipocytes (24). On the contrary, the increase in muscle mass or muscle strength can decrease the risk of CAD (25, 26).

The incidence of sarcopenia after aortic valve replacement ranges from 21.0 to 70.2% (27). Low muscle mass is a significant predictor of increased mortality rates, prolonged hospital stays, and decreased functionality in patients after undergoing aortic valve replacement. Multiple studies have demonstrated a strong association between reduced muscle mass and higher mortality rates among post-aortic valve replacement patients (28, 29).

PAD leads to reduced blood flow in the lower limbs, which restricts the supply of energy and oxygen to the leg muscles, thereby affecting their function and quality. This may further result in sarcopenia. The incidence of sarcopenia in patients with atherosclerotic occlusive disease of the lower extremities can be as high as 35% (30). Patients with PAD who have sarcopenia experience significantly higher rates of mortality and amputation compared to those without sarcopenia (31).

## 3 Diagnosis methods for sarcopenia in cardiovascular diseases

The most widely used diagnostic criteria for sarcopenia currently are the consensus revised by EWGSOP in 2018 (32), which refer to low muscle mass accompanied by poor muscle strength or physical performance. Muscle mass was measured using either BIA or DXA, muscle strength was assessed through grip strength, and physical performance was evaluated by gait speed. By using DXA to measure the appendicular skeletal muscle mass (ASM) and converting it through a formula, appendicular skeletal muscle mass index (ASMI) is calculated. The cutoff values for ASMI are <7.0 kg/m^2^ for males and < 6.0 kg/m^2^ for females. The cutoff values for grip strength are <27 kg for males and < 16 kg for females, while the cutoff value for 6 m gait speed is ≤0.8 m/s for both males and females. The more suitable diagnostic criteria for sarcopenia in Asian populations proposed by AWGS (2019) include the following cutoff values: ASMI for muscle mass - DXA: males <7.0 kg/m^2^, females <5.4 kg/m^2^; BIA: males <7.0 kg/m^2^, females <5.7 kg/m^2^; grip strength - males <28 kg, females <18 kg; and usual gait speed - both males and females at a cutoff value of 1.0 m/s (3).

### 3.1 Physical methods

The SARC-F questionnaire, developed by Malmstrom et al. (33) is a screening tool for sarcopenia that consists of five items: walking ability, rising from a chair, stair climbing and experiences with falls. A score of ≥4 indicates a positive screening result for sarcopenia and predicts a poor prognosis. The SARC-F questionnaire demonstrated low sensitivity and high specificity in diagnosing sarcopenia among elderly individuals residing in the community (34), suggesting that this questionnaire may not be effective for early screening of sarcopenia. The greater value of this questionnaire may lie in predicting prognosis, as the SARC-F questionnaire can effectively screen for sarcopenia in patients with acute and chronic CVD and serve as a predictive factor for adverse outcomes (35, 36). Studies have suggested using a cutoff score of ≥2 on the SARC-F questionnaire to diagnose sarcopenia in CVD patients, aiming to improve its sensitivity (with sensitivities of 0.635 for males and 0.758 for females) (37).

The Ishii score was first proposed by Shinya Ishii based on the two-step method recommended by EWGSOP (38). A scoring table was developed to assess the risk of sarcopenia in older adults living in the community. The final model includes three variables: age, grip strength, and calf circumference, with a threshold value for sarcopenia set at 120 for females and 105 for males in the elderly community population. However, the effectiveness of this rating in patients with CVD remains uncertain. A post-analysis provided the critical values of Ishii score for predicting sarcopenia in HF patients. For females, the value was 165 with a sensitivity of 70.9% and specificity of 68.5%. For males, the value was 141 with a sensitivity of 88.4% and specificity of 69.7% (39). The higher sensitivity suggests that this questionnaire is beneficial for early screening of sarcopenia, thereby guiding early clinical intervention to delay disease progression. In addition, in patients with HF, mid-upper arm circumference and arm muscle circumference may be more reliable than calf circumference as a variable (40), possibly due to the presence of lower limb edema in HF patients.

Barbosa-Silva et al. combined the SARC-F questionnaire with calf circumference to create SARC-CalF, which addresses the lack of muscle mass assessment in SARC-F (41). Studies have been conducted on patients with hemodialysis and type 2 diabetes (42, 43). Further research is needed to determine the effectiveness of the SARC-CalF questionnaire for CVD patients.

Research has shown that the ratio of serum creatinine (Cr) to cystatin C (CysC) can be utilized in diagnosing sarcopenia. Cr, a derivative of skeletal muscle protein phosphocreatine, is excreted by the kidneys and serves as a routine serum marker for estimating GFR. Its value reflects both muscle mass and kidney function (44). In contrast, Cys is produced by all nucleated cells with limited impact on its levels compared to Cr due to its dependence primarily on renal function (45). Considering the differences in metabolism between these two biomarkers, Cr/CysC is considered as a promising alternative marker for muscle mass (46, 47). Kashani et al. (46) initially coined the term sarcopenia index (SI) to refer to the product of Scr/CysC×100, and they validated a linear positive correlation between SI and muscle mass measured by abdominal CT scan at L4 level. Shi et al. (48) provided the optimal cutoff values for diagnosing low skeletal muscle mass based on the Cr/CysC ratio (men <1.0, women <0.8). They further developed an estimated ASM equation that incorporates age, gender, height, weight, Cr, and CysC parameters. Compared to actual ASM measured by DXA, this equation demonstrates a sensitivity and specificity of over 80% in diagnosing low muscle mass (49). In patients with HF, aortic valve replacement surgery, and hypertension, SI can also serve as an alternative indicator for muscle mass (50–52). A retrospective study established a model that combines human measurement data and SI to estimate ASMI in HF patients and obtained cutoff values (male ASMI <7.0 kg/m^2^, female <5.4 kg/m^2^) (53). The model corrects the influence of edema on simple human measurement models (including age, weight, and height), providing higher reliability and accuracy.

If the screening tool suggests probable sarcopenia, the next recommended step is assessing muscle strength as the primary parameter of sarcopenia. EWGSOP2 recommends using handgrip strength or 5-time chair stand test to indicate skeletal muscle strength.

### 3.2 The visualization methods

#### 3.2.1 Computed tomography

The cross-sectional area analysis of CT is utilized for evaluating the cross-sectional area (CSA) of muscles through both axial CT scans (abdomen or mid-thigh) and peripheral CT scans (lower leg) (54). Currently, measuring the CSA of the skeletal muscle using CT images at the level of the third lumbar vertebra (L3) is considered the gold standard for assessing muscle mass. Therefore, in many studies, a decrease in muscle mass is defined by the CSA of muscles at the L3 level (55). However, in practical clinical work, since routine imaging of many patients does not include the L3 level, alternative skeletal muscle indices from other levels need to be used as substitutes. For healthy subjects, Moon et al. first reported gender-specific cutoff values for diagnosing sarcopenia in most Asians using the area of the fourth thoracic (T4) level muscle group: 100.06 cm^2^ for males and 66.93 cm^2^ for females (56). Additionally, recent studies have also suggested that levels such as the first lumbar vertebrae (L1), fourth lumbar vertebrae (L4), twelfth thoracic vertebrae (T12), and upper thigh can be used to assess sarcopenia (57–59). CT-derived quantitative body composition analysis methods and cutoff values vary for the assessment of sarcopenia in patients with different CVD. A prospective study has confirmed that low skeletal muscle mass, identified by CT at the level of the first lumbar vertebra (L1), is an independent predictor of adverse prognosis in patients with CAD. Furthermore, a specific diagnostic threshold applicable to East Asian populations has been introduced at the L1 level, with a skeletal muscle mass index (SMI) of 31.00 cm^2^/m^2^ for males and 25.00 cm^2^/m^2^ for females (60). The unilateral pectoralis muscle mass indexed to body surface area (PMI) and attenuation (approximated by mean Hounsfield units; PHUm) can quantify muscle loss in HF patients (61). Additionally, the CT-derived fatty muscle fraction (FMF) is a potential new biomarker for sarcopenia, providing additional information for risk stratification in patients undergoing transcatheter aortic valve replacement (62). The subcutaneous fat index (SFI) and SMI, measured at the L3 vertebral level, can serve as biomarkers for sarcopenia in patients undergoing endovascular aneurysm repair surgery (63). Accurate manual segmentation of different body compositions is of great significance for measuring body composition, but this task takes 10 to 30 min. Therefore, AI-based image analysis techniques, such as automated deep learning technology, have been developed for quantitative assessment of body composition (such as muscles, visceral fat, subcutaneous fat, etc.) (64–67). Compared to manual segmentation, AI-based image analysis significantly reduces the required time and offers higher effectiveness and reliability. Weston et al. developed a fully automated technique using deep convolutional neural networks for abdominal segmentation, which can achieve even higher accuracy than manual segmentation (68). Subsequently, LEE et al. utilized this technique to evaluate the level of the L3 skeletal muscle area (SMA) in patients after aortic valve replacement surgery and found that low SMA was significantly associated with poor prognosis. They also obtained gender-specific Z-score cutoff values for male and female SMAs at 41.2 cm^2^/m^2^ and 33.0 cm^2^/m^2^, respectively (69). In general, CT can directly reflect the muscle mass of specific parts of the human body and, by calculating muscle density, it can more accurately evaluate the quality and structural characteristics of muscles. Therefore, CT is considered to be the most accurate method for assessing muscle mass (70). However, due to difficulties in performing CT measurements, relatively high costs, certain radiation exposure risks, lack of normal reference ranges and diagnostic thresholds at present, it is not suitable for screening large samples of populations and thus has not been widely used in clinical practice.

#### 3.2.2 Magnetic resonance imaging

MRI is also a measurement method that can accurately assess muscle mass. Due to the presence of varying degrees of decreased size and quantity of type II muscle fibers, as well as intramuscular and intermuscular fat infiltration in patients with sarcopenia (71), water-fat separation MRI based on Dixon imaging technology achieves high soft tissue contrast, allowing for precise measurement of muscle tissue and fat infiltration (72). Therefore, compared to BIA, MRI can accurately identify tissues such as muscles, tendons, fibers, and fats without being affected by intramuscular fat. With the advancement of technology, not only conventional MRI sequences such as T1 but also techniques like diffusion tensor imaging, ultra-short echo time imaging, T2 mapping, and diffusion-weighted imaging are gradually being applied for evaluating muscle status (73–75). The CSA of muscle measured using MRI at the L3 can effectively predict total body muscle mass (76). Kiefer et al. proposed the use of standardized manual segmentation-algorithm for quantitatively evaluating total muscle mass and fat-free muscle mass, calculating the indices of total abdominal muscle mass and fat-free abdominal muscle mass to assess muscle quality (77). Recently, studies have found that measuring the chest muscles using cardiac magnetic resonance imaging (CMR) may hold potential in assessing sarcopenia. One study discovered that unilateral chest muscle measurement under CMR demonstrated a strong predictive value for postoperative mortality among patients who underwent surgical aortic valve replacement (78). Furthermore, the utilization of bilateral SMI based on heart MRI has emerged as a novel approach to evaluate sarcopenia in HF patients (79). Consequently, opportunistic screening for sarcopenia becomes feasible during cardiac MRI examinations. However, the manual segmentation of muscles based on MRI is also time-consuming and may take several days, which limits its application and promotion in clinical work. Therefore, we need to explore new probabilistic methods such as deep learning for muscle segmentation. The previous studies have developed automatic segmentation technology for the three-dimensional structure of the quadriceps, enabling quantitative evaluation of quadriceps volume (80). Additionally, a fast whole-body MRI method has been developed to automatically quantify total skeletal muscle volume and volumes of individual muscle groups (81). However, there is currently a lack of widely available segmented MRI datasets for skeletal muscles, and the use of artificial intelligence-based MRI techniques for assessing muscle mass reduction in sarcopenia remains limited. It should be noted that MRI is expensive and time-consuming for whole-body scans, lacks normal reference ranges and diagnostic thresholds, and its application in populations is greatly restricted due to limitations on subjects with metallic implants (82).

#### 3.2.3 Ultrasound

The ultrasound can assess muscle condition by measuring parameters such as muscle thickness, cross-sectional area, muscle volume, muscle fiber length, pennation angle, echogenicity, and muscle hardness (83). Among them, quadriceps muscle imaging has been proven to be a reliable predictor of overall skeletal muscle quality. Previous studies have confirmed the diagnostic value of ultrasound quadriceps muscle imaging for secondary sarcopenia in diseases such as chronic obstructive pulmonary disease, Parkinson’s disease, liver cirrhosis, and stroke (84–87). A study has found that the difference in cross-sectional area (ΔCSA) and shear wave elastography (ΔSWE) between the contracted and relaxed states of the rectus femoris muscle can serve as an independent predictor for sarcopenia in elderly patients with type 2 diabetes. Furthermore, a model was established that combines age, ΔCSA, and ΔSWE, which demonstrated a sensitivity and specificity of 83.3% (88). However, there is currently limited research on the ultrasound diagnosis of cardiovascular disease combined with sarcopenia. A cross-sectional study focused on elderly HF patients found that echo intensity of the quadriceps femoris and subcutaneous fat thickness in the non-contractile state were associated with muscle strength in elderly HF patients (89). Taira et al. used ultrasound to measure the anterior femoral muscle thickness of 1,075 patients with CVD, using the diagnostic criteria of AWGS as the gold standard. They found a cutoff value of 2.425 cm for males, with a sensitivity and specificity of 68.5 and 77.6%, respectively; for females, the cutoff value was 1.995 cm, with a sensitivity and specificity of 70.5 and 66.0%, respectively (90). There is still significant research potential for diagnosing CVD combined with sarcopenia using ultrasound examination. Ultrasound examination offers strong portability, relative affordability, and no radiation exposure, making it suitable for clinical or community screening and follow-up. It can also be performed at the bedside. However, obtaining ultrasound images and interpreting results rely more on the technical skills of operators.

#### 3.2.4 Dual-energy X-ray absorptiometry

Baumgartner et al. developed a diagnostic method that utilizes dual DXA to assess the SMI (91). The DXA scan is a non-invasive, easy-to-operate, cost-effective method with relatively low radiation dose for measuring muscle mass. It accurately distinguishes between whole-body and local muscles, fat, and bones, making it widely used in clinical practice. However, DXA allows for a whole-body estimation of lean mass, which measurement is actually an estimation of all non-fat/non-bone tissues. In addition, it is worth noting that DXA measurements may be influenced by the patient’s hydration status (92). This effect is particularly evident in the measurement of lower limb skeletal muscle mass in HF patients due to fluid retention in the lower limbs (93). In contrast, CT and MRI show high accuracy in the assessment of muscle and fat CSA/volume with the segmentation of muscles on cross-sectional images. CT can measure muscle size and attenuation in specific districts. MRI allows measuring the amount of muscle and fat tissue due to its high contrast resolution and multiparametricity.

#### 3.2.5 Bioelectrical impedance analysis

BIA is a widely used non-invasive method for measuring body composition. Its principle involves using surface electrodes to record the different electrical resistances of various tissues and then utilizing image reconstruction techniques to measure muscle mass (94). Consensus guidelines published by AWGS and EWGSOP have provided recommended cutoff values for diagnosing muscle loss based on BIA-measured ASM. However, the currently available BIA prediction models have poor accuracy, and their measurement methods are easily influenced by factors such as body water content and electrolyte imbalances. Due to the presence of varying degrees of edema in HF patients, there is a significant margin of error when assessing muscle mass using BIA (95). BIA is also influenced by obesity, often leading to an overestimation of muscle mass in obese patients (77). For individuals with sarcopenic obesity, the muscle-to-fat ratio measured by BIA may be a more appropriate biomarker for defining and diagnosing sarcopenia (96). In addition to ASM, phase angle (PA) is a parameter derived from BIA that predicts various clinical outcomes and mortality rates of diseases (97). It can be obtained by measuring the ratio of reactance (Xc) to resistance (R) (PA = arctangent Xc/R), providing information on muscle mass and function. The magnitude of the PA value mainly depends on the size of cell membrane capacitance. A low PA value indicates lower cell membrane capacitance and poorer cell membrane structure and function (98). A meta-analysis, using the European Consensus 2010/2019 and Asian Consensus 2014 diagnostic criteria for sarcopenia, determined that the cut-off range for diagnosing sarcopenia with phase angle was between 3.55°to 5.05° (99). The phase angle cut-off value for sarcopenia in elderly HF patients was found to be 5.45°, with a sensitivity of 76% and specificity of 71% (100). Although BIA is non-invasive and easy to use, it cannot be used on individuals with pacemakers due to the weak electrical current employed (101).

### 3.3 Molecular level

Inflammatory factors play a crucial role in the occurrence and development of sarcopenia. Inflammaging, characterized by a low-grade chronic inflammatory state caused by immune system damage that occurs with age, is the main mechanism involved. It includes immunosenescence, increased secretion of inflammatory mediators from visceral fat inflammation, dysbiosis of the microbiota, and accumulation of senescent cells. These mechanisms ultimately lead to the infiltration of neutrophils and monocytes/macrophages into adipose tissue and other tissues, resulting in excessive secretion of pro-inflammatory cytokines (102, 103). As individuals age, there is a gradual increase in the expression of pro-inflammatory cytokines. Inflammatory factors inhibit myoblast fusion, stimulate excessive production of reactive oxygen species by mitochondria, activate the ubiquitin-proteasome system, induce autophagy and apoptosis in skeletal muscle cells, accelerate skeletal muscle protein degradation. This ultimately leads to the occurrence and development of sarcopenia (104, 105). Among them, the elevation of inflammatory factors such as tumor necrosis factor-*α* (TNF-α), C-reactive protein (CRP), and IL-6 levels is associated with the decline in skeletal muscle strength and quality (106). In HF patients with sarcopenia, IL-6 and CRP are elevated (107, 108). TNF-α activates the transcription factor nuclear factor-κB, thereby increasing protein degradation and promoting muscle atrophy (109). Prolonged high levels of IL-6 can contribute to muscle wasting in conjunction with other mediators, while CRP is associated with insulin resistance, inhibiting muscle function and leading to decreased strength (110).

Homocysteine (Hcy) is a sulfur-containing amino acid that plays an important role in the remethylation and transsulfuration pathways in the human body (111). Elevated blood Hcy levels can lead to oxidative stress, protein aggregation and dysfunction, cell apoptosis, inflammation, mitochondrial dysfunction, resulting in reduced muscle fiber regeneration and decreased energy production. These effects partially contribute to the occurrence and development of sarcopenia (112, 113). Recent studies have shown a significant association between Hcy and decreased muscle strength, including among patients with PAD (114, 115).

miRNA-1-3p is primarily produced by skeletal muscles and regulates the proliferation and differentiation of muscle cells (116). miR-1-3p is also a biomarker associated with the pathogenesis of HF (117), which leads to skeletal muscle damage and related cell death, resulting in passive release of miR-1-3p into the systemic circulation. The study found a significant correlation between the expression of miRNA-1-3p and the activation of the Akt/mTOR pathway (118). The levels of miRNA-1-3p in HF patients with sarcopenia were significantly higher than those without sarcopenia, and there was a strong correlation between miRNA-1-3p expression and both ASMI as well as grip strength. The cutoff value for predicting muscle wasting using miR-1-3p was 1.01, with a sensitivity of 75.0% and specificity of 62.5%. These findings suggest that this small molecule can serve as a predictive marker for sarcopenia in HF patients.

Research has found that the expression of HIF-1α and pax7 is significantly reduced in sarcopenia (119). HIF-1α, a major regulator of oxygen-dependent expression of several target genes involved in oxygen transport, metabolic adaptation, angiogenesis, as well as various cellular functions such as cell cycle regulation and apoptosis (120), shows significant reduction.Pax7 serves as the primary stem cell marker for satellite cells, which are regenerative cells in skeletal muscle. These cells proliferate in response to physiological stimulation, injury, and degenerative diseases, resulting in a significant increase in myogenic cell proliferation. Subsequently, these myogenic cells differentiate into muscle fibers to facilitate skeletal muscle regeneration (121). This indicates that HIF-1α and Pax7 can be utilized for diagnosing sarcopenia.

Wnt signaling is involved in muscle development, muscle regeneration, and stem cell renewal during processes of muscle atrophy and muscle wasting. Upregulation of Wnt signaling during aging can inhibit myogenesis and promote sarcopenia (122). A randomized controlled study on HF patients found a significant correlation between hand grip strength and three biomarkers of Wnt signaling: dickkopf-3 (Dkk-3), sterol regulatory element-binding protein-1 (SREBP1), and dickkopf-1 (Dkk-1) (123). This suggests that they have significant potential as plasma biomarkers for assessing sarcopenia in HF patients.

Some messenger RNAs, such as HERC5, S100A11, and FLNA, have also been shown to serve as potential biomarkers for sarcopenia (124). Serum meteorin-like protein (Metrnl), a novel myokine with protective effects against CVD, has been found to be associated with sarcopenia in elderly patients with HF (125). The phylum Synergistetes has also been identified as a potential biomarker for sarcopenia in HF patients (126). In elderly patients with CVD, the triglyceride-to-high-density lipoprotein cholesterol ratio (TG/HDL-C) is negatively correlated with relative grip strength (127), suggesting that this ratio may be used to evaluate sarcopenia in CVD patients. However, these serum markers are not specific for diagnosing sarcopenia.

## 4 Summary and future prospects

Nowadays, the attention to sarcopenia is increasing year by year, and different countries have different diagnostic methods and thresholds for different populations with sarcopenia. Currently, comprehensive diagnosis mainly relies on assessing muscle mass, muscle strength, and physical function. Finding a simple and reliable alternative diagnostic indicator remains an urgent problem for researchers in the field of sarcopenia. Physical methods are simple and feasible, but they have low sensitivity and are not conducive to early screening. Among them, the estimation equation demonstrates high sensitivity and specificity, making it a promising new method for assessing muscle mass. In the future, visualization will become a trend. CT and MRI are often used in clinical examinations, so there may be an opportunity to apply CT and MRI imaging for screening CVD in the clinical diagnosis of sarcopenia. However, there is currently a lack of standardized diagnostic protocols, and manual segmentation is time-consuming. Therefore, research on AI-based fully automated segmentation methods may be the focus. In addition, three-dimensional imaging techniques based on CT and MRI can directly assess the volume of skeletal muscles, which may more accurately represent muscle mass than CSA of muscle. Ultrasound examination can be used as a dynamic monitoring method in clinical practice; however, there is currently a lack of evaluation for other muscle groups besides the quadriceps femoris. Apart from two-dimensional ultrasound imaging techniques, other ultrasound technologies such as shear wave elastography are also worth further research. Currently, there is a lack of specificity in serum biomarkers for diagnosing sarcopenia, and more high-quality studies are needed to explore and identify a specific serum biomarker as a diagnostic indicator.
